# Core-Shell Microspheres Prepared Using Coaxial Electrostatic Spray for Local Chemotherapy of Solid Tumors

**DOI:** 10.3390/pharmaceutics16010045

**Published:** 2023-12-28

**Authors:** Xiaowei Zhang, Rundong Zhu, Xingzhi Wang, Hao Wang, Zushun Xu, Yongan Wang, Dongqin Quan, Liao Shen

**Affiliations:** 1 State Key Laboratory of Toxicology and Medical Countermeasures, Beijing Institute of Pharmacology and Toxicology, Beijing 100850, China; a836446450@163.com (X.Z.); zhurundongleon@163.com (R.Z.); w12555596749@163.com (X.W.); wang91467@163.com (H.W.); yonganw@126.com (Y.W.); 2 School of Materials Science and Engineering, Hubei University, Wuhan 430062, China; a163xuzushun@163.com

**Keywords:** chemotherapy, controlled drug-release, microspheres, electrostatic spray

## Abstract

Local chemotherapy is an alternative therapeutic strategy that involves direct delivery of drugs to the tumor site. This approach avoids adverse reactions caused by the systemic distribution of drugs and enhances the tumor-suppressing effect by concentrating the drugs at the tumor site. Drug-loaded microspheres are injectable sustained-release drug carriers that are highly suitable for local chemotherapy. However, a complex preparation process is one of the main technical difficulties limiting the development of microsphere formulations. In this study, core-shell structured microspheres loaded with paclitaxel (PTX; with a core-shell structure, calcium alginate outer layer, and a poly (lactic acid-co-glycolic acid) copolymer inner layer, denoted as PTX-CA/PLGA-MS) were prepared using coaxial electrostatic spray technology and evaluated in vitro and in vivo. PTX-CA/PLGA-MS exhibited a two-stage drug release profile and enhanced anti-tumor effect in animal tumor models. Importantly, the preparation method reported in this study is simple and reduces the amount of organic solvent(s) used substantially.

## 1. Introduction

Cancers are a major clinical challenge, and significantly enhance the morbidity and mortality rates of cancer patients [1]. In the early stages of cancer, patients usually do not show discernible symptoms, with most already in the middle to late stages of the disease at diagnosis; therefore, the optimal time for surgical resection is often missed [2]. Such patients usually require radiotherapy and systemic chemotherapy to inhibit tumor growth. For some solid tumors, local chemotherapy involves the direct delivery of chemotherapeutic drugs via injection or other administration methods [3]. Compared with systemic chemotherapy, local chemotherapy allows chemotherapeutic drug concentration to build up at the tumor site while avoiding the toxic effects of drugs on normal tissues and organs, thereby reducing adverse reactions in patients [4,5]. To this end, local chemotherapy is an alternative treatment for solid tumors that are difficult to treat using surgical resection. However, most anti-cancer therapeutics are small-molecule drugs that can “spill out” even after direct local injection [6]. Therefore, the effects of local drug administration may not differ substantially from those of intravenous or oral administration [7].

Implantable drug-releasing carriers such as microspheres (MS) and rods can effectively limit drug localization to the implanted area, thereby increasing the drug concentration at the target site [8,9]. They are thus highly suitable for local chemotherapy of tumors [10]. Among these carriers, drug-loaded MS have become the most commonly used carriers because of their excellent biocompatibility and sustained release properties [11]. MS are small spheres made of polymer materials that typically range from a few to several hundred micrometers in diameter [12]. They can be used for controlled drug release while protecting the bioactive molecules [13]. However, the preparation process for MS is usually complex and requires customization and continuous improvement of the equipment. Additionally, the preparation conditions optimized from small-scale experiments are rarely directly applicable for large-scale production, which can lead to failed scale-up production and substantial financial waste [14,15].

The electrospray method is a new method for MS preparation [16]. In this process, the fundamental polymer material for the production of MS is initially dissolved in a suitable solvent to generate a polymer solution [17]. Subsequently, using a microliquid delivery pump, the solution is transported along a piping system to a nozzle, and an electric field is applied [18]. Under the action of an external electric field, the polymer solution at the nozzle tip generates an electrostatic force [19]. When the electrostatic force surpasses the surface tension of the droplets, the solution is ejected from the nozzle tip as charged droplets, which disperse owing to Coulomb repulsion and subsequently enter a collection device [20]. Finally, the droplets undergo drying and solidification to form MS [21]. Compared with current preparation methods of MS, electrostatic spraying involves fewer process parameters that are easier to control, resulting in higher process optimization efficiency and reduced use of organic solvents during the preparation process, making it easier to control the final solvent residue [22]. Furthermore, by optimizing the liquid delivery system, it is easier to achieve continuous production of MS at lower costs for scaled-up production [23].

Therefore, in this study, coaxial electrospray was employed to prepare paclitaxel (PTX)-loaded calcium alginate-poly (lactic acid-co-glycolic acid) (PLGA) composite MS. Paclitaxel, a commonly used broad-spectrum anti-cancer drug, was used as a model drug and coaxial electrospray as an improved electrostatic spraying technique. The PTX containing MS (termed, PTX-CA/PLGA-MS) had a core-shell structure, calcium alginate outer layer, and a poly (lactic acid-co-glycolic acid) copolymer inner layer. The outer calcium alginate layer had a loose skeleton structure and could swell rapidly after absorbing water. Therefore, soon after injection of the MS, the PTX on the outer layer can rapidly release into the tumor tissue, thus achieving a high drug concentration in a short time to swiftly kill tumor cells. The PLGA core of the MS is a relatively compact structure; therefore, PTX could release at a slow rate from the MS, maintaining drug concentration in the tumor for a certain period of time to achieve continuous killing of tumor cells. PTX-CA/PLGA-MS could simultaneously achieve immediate/slow release of chemotherapeutics, enhancing local chemotherapy as well as effectively reducing dosing frequency. Furthermore, administration of PTX-CA/PLGA-MS could reduce drug spread from the tumor region, in turn preventing serious adverse reactions. Owing to this, the safety of intratumoral injection of chemotherapy drugs is significantly improved with this delivery technology. Herein, both in vitro and in vivo evaluations were implied to assess the therapeutic potential of PTX-CA/PLGA-MS.

## 2. Materials and Methods

### 2.1. Materials

Sodium alginate (CP, viscosity: 200 ± 20 mPa·s) was purchased from Shanghai Macklin Biochemical Co., Ltd. (Shanghai, China). Egg Phosphatidyl Choline (EPC) was purchased from Lipoid GmbH (Ludwigshafen, Germany). Fluorescein sodium, CCK-8 kit, and DIR were purchased from Yeasen Biotechnology (Shanghai) Co., Ltd. (Shanghai, China). Mannitol was acquired from Shanghai Aladdin Biochemical Technology Co., Ltd. (Shanghai, China). Anhydrous calcium chloride, sodium dodecyl sulfate, and dichloromethane were purchased from Anhui Senrise Technology Co., Ltd. (Shanghai, China). Poly lactic-co-glycolic acid (molecular weight 24,000–38,000) was purchased from Xi’an ruixi Biological Technology Co., Ltd. (Xi’an, China). Methanol, absolute ethanol, and acetonitrile were purchased from Thermo Fisher Scientific (Shanghai, China). Fetal bovine serum (FBS) was purchased from Gibco (Grand Island, NY, USA). Antibiotics (100 U/mL penicillin and 100 U/mL streptomycin) and non-essential amino acids (NEAA) were purchased from Sigma Aldrich (St. Louis, MO, USA). All other chemicals used in this study were of analytical or high-performance liquid chromatography (HPLC) grade.

### 2.2. Preparation of PTX-CA/PLGA-MS

PTX liposomes were prepared using the ethanol injection method, where 360.0 mg EPC was dissolved in 2.0 mL ethanol to form a homogeneous solution, followed by the addition of 40.0 mg PTX. This solution was then added dropwise to 18.0 mL deionized water, and a film extrusion method was used to obtain the PTX liposome solution. Then, 2.0 mL PTX liposomes were mixed with 2.0 mL of 1% sodium alginate solution (containing 2.5% sodium dodecyl sulfate and 17.5% mannitol) to form the outer layer of MS prepared using coaxial electrostatic spraying. Poly lactic acid-co-glycolic acid (0.24 g) and PTX (24.0 mg) were dissolved in dichloromethane (3 mL) to form the internal oil phase. Next, 3% calcium chloride solution was used as the receiving solution. For preparation, the electrospray voltage was adjusted from 6.0 to 10.0 kV, and the injection rates of the outer phase and inner phase were 4 mL/h and 3 mL/h, respectively. All MS were collected and washed with deionized water to remove calcium chloride from the solution, and then subjected to freeze-drying to remove water and dichloromethane.

### 2.3. Characterization of PTX-CA/PLGA-MS

#### 2.3.1. Appearance and Morphology

Suitable amounts of freshly prepared and lyophilized PTX-CA/PLGA-MS were placed on glass slides, and the samples were observed and photographed under an optical microscope. A scanning electron microscope (SEM, JEOL 5800LV, Tokyo, Japan) was used to observe the morphology of PTX-CA/PLGA-MS. Prior to the analysis, the lyophilized PTX-CA/PLGA-MS powder was coated with a thin layer of gold using a sputter-coating device.

#### 2.3.2. Particle Size and Structure

The size distribution of PTX-CA/PLGA-MS was determined using a Mastersizer 2000 (Shanghai Spectrum Instruments System Co., Ltd., Shanghai, China). A laser confocal microscope (LEXT-OLS510, Olympus, Tokyo, Japan) was used to observe the core-shell structure of PTX-CA/PLGA-MS. The inner and outer layers of PTX-CA/PLGA-MS were labeled with appropriate amounts of DIR and sodium fluorescein, respectively.

### 2.4. In Vitro Drug Release

In vitro PTX release was assessed using a dialysis-based method. PTX-CA-MS, PTX-PLGA-MS, and PTX-CA/PLGA-MS were placed into dialysis bags and dispersed in 10 mL phosphate buffer saline (PBS) solution (pH 5.0) containing 1% (*w*/*v*) Tween 80, and incubated at 37 ± 1 °C at a stirring speed of 100 rpm. At predetermined time intervals, buffer samples were withdrawn and replaced with an equal volume of fresh buffer. The PTX content was determined by HPLC.

(1) $$\mathrm{Cumulative}\;\mathrm{release}\;=(\frac{v_{0}C_{n}+v_{s}\sum_{1}^{n-1}\;C_{t}}{m_{0}})\times 100\%$$

where m_0_ is the total mass of the drug in PTX-CA-MS, PTX-PLGA-MS, and PTX-CA/PLGA-MS. v_0_ is the initial volume, v_s_ is the sampling volume, c_n_ and c_t_ are the drug concentrations, and t and n are the sampling times. All assays were performed in triplicate in the dark.

### 2.5. In Vitro Cytotoxicity Evaluation

The effect of blank MS on HepG2 cell viability was evaluated using the CCK-8 assay. The cells were grown to a density of 8 × 10^3^ cells/mL and seeded into a 96-well plate (200.0 μL/well). The plates were incubated at 25 °C for 24 h to allow the cells to adhere. The effects of CA-MS, PLGA-MS, CA/PLGA-MS, PTX solution, PTX-PLGA-MS, and PTX-CA/PLGA-MS were investigated at three concentration levels (L: low dosage; M: medium dosage; H: high dosage). For CA-MS, PLGA-MS, and CA/PLGA-MS, 15.0 mg (L), 30.0 mg (M), and 45 mg (H) of MS were dispersed in 50 mL PBS (pH 7.4) and incubated at 37 ± 1 °C for 24 h at a stirring speed of 100 rpm. For PTX-CA-MS, PTX-PLGA-MS, and PTX-CA/PLGA-MS, 1.5 mg (L), 3.0 mg (M), and 4.5 mg (H) of MS were dispersed in 50 mL PBS (pH 7.4) and incubated at 37 ± 1 °C for 24 h at a stirring speed of 100 rpm. PTX solution at 100 (L), 200 (M), and 300 ng/mL (H) was used as the positive control. Then, 10 µL of leachate from each group of MS was added to each well. The plate was then incubated at 37 °C (5% CO_2_) for 24, 48, and 72 h, and cell viability was determined using CCK-8 kits with a microplate reader (iMark, Bio-Rad Laboratories, Benicia, CA, USA) at 450 nm. Cell viability was calculated using the following formula:

(2) $$\mathrm{Cell}\;\mathrm{Viability}\;=\frac{OD_{\mathrm{test}\;}-OD_{\mathrm{blank}\;}}{OD_{\mathrm{control}\;}-OD_{\mathrm{blank}\;}}*100\%$$

(3) $$\mathrm{Inhibition}\;\mathrm{Rate}=(1-\frac{OD_{\mathrm{test}\;}-OD_{\mathrm{blank}\;}}{OD_{\mathrm{control}\;}-OD_{\mathrm{blank}\;}})*100\%$$

where, OD_blank_ is the optical density of the blank well (PBS and CCK-8 reagent alone), OD_test_ is the optical density of the test group, and OD_control_ is the optical density of the control group.

### 2.6. In Vivo Anti-Tumor Activity Evaluation

A total of 24 female BALB/c mice (aged 6~8 weeks, obtained from Beijing Vital River Laboratory Animal Technology Co., Ltd., Beijing, China) carrying H22 tumor cells were randomly divided into four groups, namely saline (control), PTX, PTX-PLGA-MS, and PTX-CA/PLGA-MS. Saline or PTX solutions (200 µg/kg) were injected intratumorally every 2 d in the saline or PTX groups, respectively. In this group, PTX solution was injected intratumorally every 2 days, mainly to avoid accidental death of experimental animals caused by excessive drug dosage, and this medication regimen was also similar to the actual clinical medication method. Mice in experimental groups of MS received a single intratumoral injection of PTX-PLGA-MS or PTX-CA/PLGA-MS PTX at a dose of 1 mg/kg at the beginning of the experiment. On day 14, the mice were euthanized and the excised tumors were photographed and weighed. The longest (L) and shortest (W) diameters were recorded daily. The tumor volume was calculated using the following formula:

(4) $$\mathrm{Tumor}\;\mathrm{volume}\;=\frac{L\times W^{2}}{2}$$

On day 14, all mice in each group were euthanized, and all tumors were removed. The tumor tissue was immediately immersed in 10% formalin and embedded in paraffin. After slicing the paraffin-embedded specimen, hematoxylin-eosin (HE) staining was used to stain the tissue and scanned as a picture for observation. All animal experiments complied with the regulations of the Animal Care and Use Ethics Committee of the Beijing Institute of Pharmacology and Toxicology.

### 2.7. In Vivo Drug Release

Female mice (aged 6~8 weeks) bearing H22 tumors were randomly divided into three groups: PTX solution, PTX-PLGA-MS, and PTX-CA/PLGA-MS. Each group received a single intratumoral injection of (A) PTX solution, (B) PTX-PLGA-MS, or (C) PTX-CA/PLGA-MS. Tumor and surrounding tissues were collected at 2, 4, or 8 h, or 1, 5, 10, and 14 d after administration, and homogenized with 0.5 mL acetonitrile-water (50:50, *v*/*v*). Next, 50 μL internal standard solution (1 mg/mL docetaxel) was added to the mixture, followed by vortexing with 0.5 mL tert-butyl methyl ether for 3 min. The mixture was centrifuged at 5000 rpm for 5 min and the supernatant was collected. This extraction process was repeated three times and the supernatants were combined. The sample was centrifuged to produce a concentrate and dried at 4 °C. The sample was then resuspended in 0.30 mL acetonitrile-water (50:50, *v*/*v*) and then a 20 μL aliquot was injected into the HPLC system for analysis. The percentage of the released drug was calculated using the following formula:

(5) $$P\;(\%)\;=\frac{C_{0}-C}{C_{0}}\times 100\%$$

where P represents the percentage of drug residues in tumor residual MS, C (μg) represents the content of the residual drug in the injection region, and c_0_ represents the actual drug dosage (μg, calculated by PTX) that administered in the tumor region.

### 2.8. Statistical Analysis

The data are presented as mean ± standard deviation (SD). The analysis of statistical significance among various treatments was carried out using analysis of variance (ANOVA). Results with *p* < 0.05 were considered statistically significant.

## 3. Results and Discussion

### 3.1. Preparation and Characterization of PTX-CA/PLGA-MS

**Figure 1:**
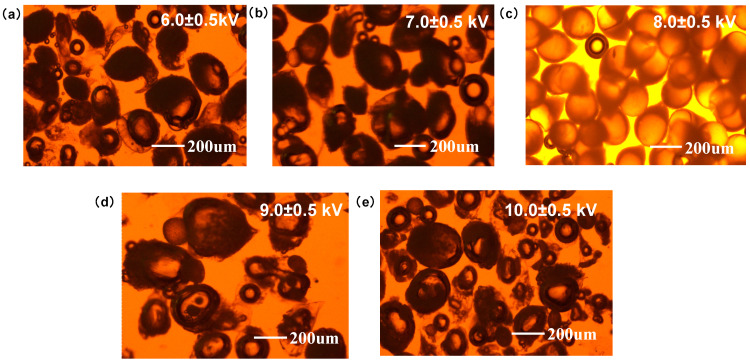
PTX-CA/PLGA-MS prepared at different voltages. MS prepared at (**a**) 6.0 ± 0.5 kV, (**b**) 7.0 ± 0.5 kV, (**c**) 8.0 ± 0.5 kV, (**d**) 9.0 ± 0.5 kV, (**e**) 10.0 ± 0.5 kV. PTX-CA/PLGA-MS: paclitaxel into MS with a core-shell structure, with the outer layer being calcium alginate and the inner layer being poly (lactic acid-co-glycolic acid) co-polymer; MS: Microspheres.

As shown in Figure 1, when the voltage was between 6.0–7.0 kV, a lower voltage was insufficient to disperse the droplets completely, leading to larger MS. In addition, the shape of the MS was irregular, with an obvious tail shape. When the voltage was between 9.0–10.0 kV, a dramatic increase in voltage resulted in jet instability, leading to multiple jets and a broadening of the particle size distribution. In addition, a high voltage broke the MS, resulting in loss of the core-shell structure, thus the distinct PLGA droplets alone were visible on photograph. Therefore, a suitable voltage is necessary for a uniform particle size distribution and the formation of an intact core-shell structure. The MS prepared at a voltage of approximately 8.0 KV had a good particle size distribution, a complete PLGA core, and a calcium alginate shell structure. The particle size of the MS without drying was approximately 180–200 μm. Compared with the commonly used methods of preparing MS such as emulsification, this method is simpler and easier to implement, requiring only a simple adjustment of the flow rate and voltage to achieve a relatively good product. Although the preparation time of microspheres is longer when using a single jetting channel, the structure of the jetting and receiving devices is relatively simple; and, theoretically, through simple equipment modification, multiple channels and continuous production can be achieved, thereby greatly improving the preparation efficiency.

**Figure 2:**
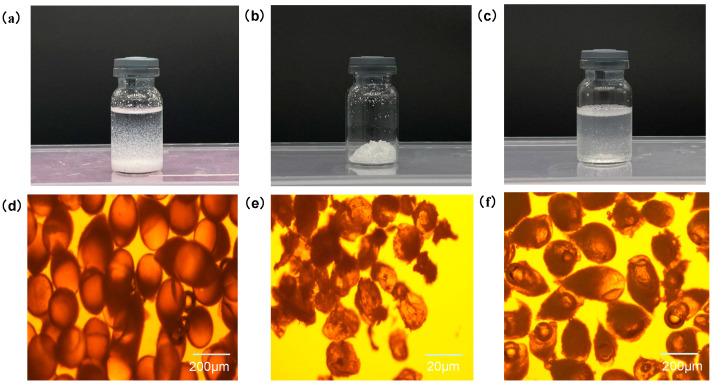
(**a**) Freshly prepared PTX-CA/PLGA-MS. (**b**) Lyophilized PTX-CA/PLGA-MS. (**c**) PTX-CA/PLGA-MS resuspended in PBS. (**d**–**f**) Optical microscopy images of freshly prepared PTX-CA/PLGA-MS, lyophilized MS, and PTX-CA/PLGA-MS suspended in PBS. PTX-CA/PLGA-MS: paclitaxel into MS with a core-shell structure, with the outer layer being calcium alginate and the inner layer being poly (lactic acid-co-glycolic acid) co-polymer; PBS: phosphate buffer saline solution; MS: microspheres.

**Figure 3:**
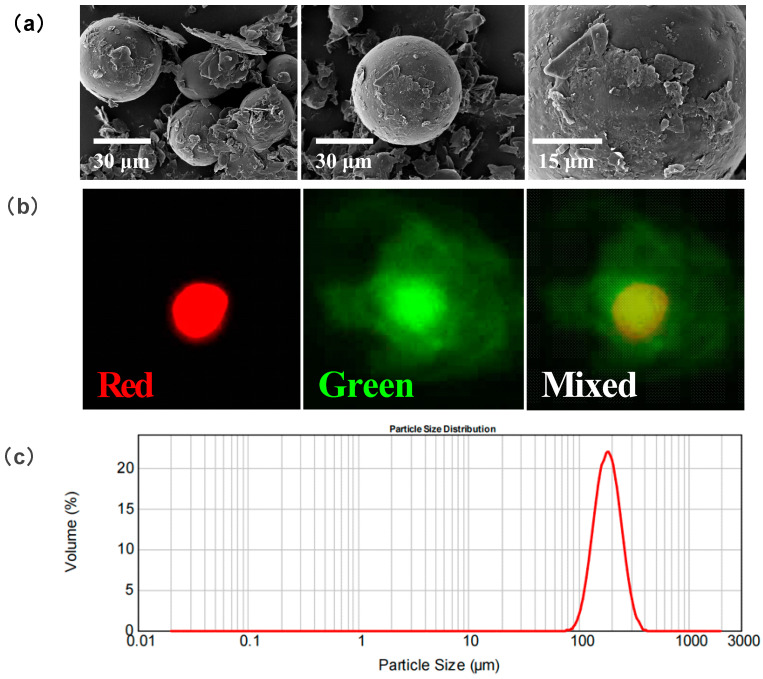
(**a**) SEM images of lyophilized PTX-CA/PLGA-MS. (**b**) Confocal microscopy imaging of lyophilized PTX-CA/PLGA-MS, green and red being the fluorescence emitted by the outer calcium alginate shell and the inner PLGA core, respectively. (**c**) Size distribution of PTX-CA/PLGA-MS. PTX-CA/PLGA-MS: paclitaxel into MS with a core-shell structure, with the outer layer being calcium alginate and the inner layer being poly (lactic acid-co-glycolic acid) copolymer; SEM: scanning electron microscope; MS: Microspheres.

Figure 2a–c shows freshly prepared PTX-CA/PLGA-MS, lyophilized MS powder, and reconstituted MS lyophilized powder suspensions, respectively. Optical microscopy results (Figure 2d–f) demonstrated that the freshly prepared PTX-CA/PLGA-MS were uniformly dispersed, nearly spherical, and of similar sizes. At this time, it can be clearly seen that the MS had an obvious bilayer structure, and the PLGA core inside existed as spherical droplets. After drying, the morphology of the PTX-CA/PLGA-MS changed to some extent; however, after rehydration and resuspension, the particle size and morphology of the MS reverted to those before drying. Since the PLGA core had completely solidified and did not contain any solvent, the particle size of the PLGA core was significantly reduced compared with that before drying. The SEM images (Figure 3a) revealed that PTX-CA/PLGA-MS was spherical or ellipsoidal in shape. Generally, dried MS are beneficial for improving drug stability and extending the shelf life of the product. Therefore, PTX-CA/PLGA-MS was suitable for freeze-drying. In the DIR and sodium fluorescein-labeled PTX-CA/PLGA-MS images shown in Figure 3b, the core and shell layers show red and green fluorescence, respectively, evidencing a clear dual-layer structure. The particle size measurement of PTX-CA/PLGA-MS showed that the average particle size was 190.763 µm; the particle size distribution is illustrated in Figure 3c.

The encapsulation and loading efficiencies of the MS were analyzed by HPLC (encapsulation efficiency (%) = actual drug content/theoretical drug content × 100%; loading efficiencies (%) = actual drug content/total weight of microspheres × 100%). The PTX-CA/PLGA-MS exhibited high entrapment efficiency and loading efficiencies of 78.31 ± 10.16% and 3.19 ± 0.41%, respectively. The core and shell drug loading accounted for 91% and 9% of the total drug mass, respectively. Notably, only a small amount of PTX was lost during MS preparation, and most of the drug was effectively encapsulated. The results indicate that this method does not cause significant losses of the drug during the preparation process and is theoretically applicable to other drugs with poor stability due to its relatively mild preparation conditions.

### 3.2. In Vitro Drug Release and Degradation

**Figure 4:**
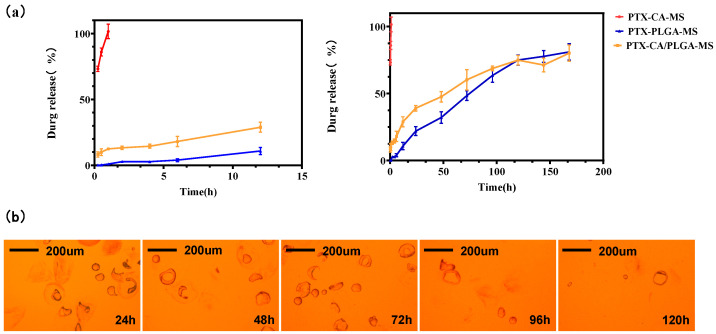
(**a**)Time-release curves for the in vitro release of PTX-CA-MS (red), PTX-PLGA-MS (blue), and PTX-CA/PLGA-MS (yellow) in PBS. Data are presented as mean ± SD (n = 3). (**b**) Optical microscopy images of the in vitro degradation of PTX-CA/PLGA-MS for 14 d. PTX-PLGA-MS: paclitaxel loaded into a single layer of poly (lactic acid-co-glycolic acid) MS; PTX-CA/PLGA-MS: paclitaxel into MS with a core-shell structure, with the outer layer of calcium alginate and the inner layer of poly (lactic acid-co-glycolic acid) copolymer; PBS: phosphate buffer solution; MS: microspheres.

Figure 4a depicts the in vitro release curves for PTX-CA-MS, PTX-PLGA-MS, and PTX-CA/PLGA-MS. PTX-CA-MS had a rapid release profile, with a cumulative release rate of 96.71 ± 5.60% within 1 h. In contrast, PTX-PLGA-MS exhibited a slow initial release, with cumulative release rates of only 4.81 ± 1.58% and 13.71 ± 3.22% within the first 6 and 12 h, respectively. The PTX-CA/PLGA-MS release profile can be divided into two stages. In the first stage, PTX-CA/PLGA-MS demonstrated rapid release, with a cumulative release rate of 14.03 ± 1.53% within the first hour. In the second stage, PTX encapsulated in the PLGA was released gradually, resulting in a cumulative release rate of 74.49 ± 5.21% after 7 d. These results demonstrate that PTX is released slowly from PTX-CA/PLGA-MS, maintaining a constant drug concentration over an extended period. This rapid release could be attributed to cracking of the calcium alginate shell, which led to the quick release of PTX. In contrast, the PLGA core structure was relatively compact and did not degrade very quickly; therefore, drug release was relatively slow. Figure 4b shows an optical image of the in vitro drug release profile of PTX-CA/PLGA-MS. During the initial 24 h, the calcium alginate shell cracked, leading to the release of PTX from the outer layer. Additionally, a portion of the inner nuclear layer of PLGA was exposed. Between 24 and 48 h, the outer layer was mostly decomposed, and the inner PLGA layer began to disintegrate, resulting in the release of PTX from the nuclear layer. After 120 h, PLGA was almost completely degraded, showing approximately complete drug release. It should be noted that in the in vitro simulated release experiment, in order to make PTX reach the leakage condition, the simulation medium contained 1% Tween 80 as a surfactant; therefore, the degradation rate of MS was significantly faster than the actual degradation rate in vivo.

### 3.3. In Vitro Cytotoxicity Analysis

**Figure 5:**
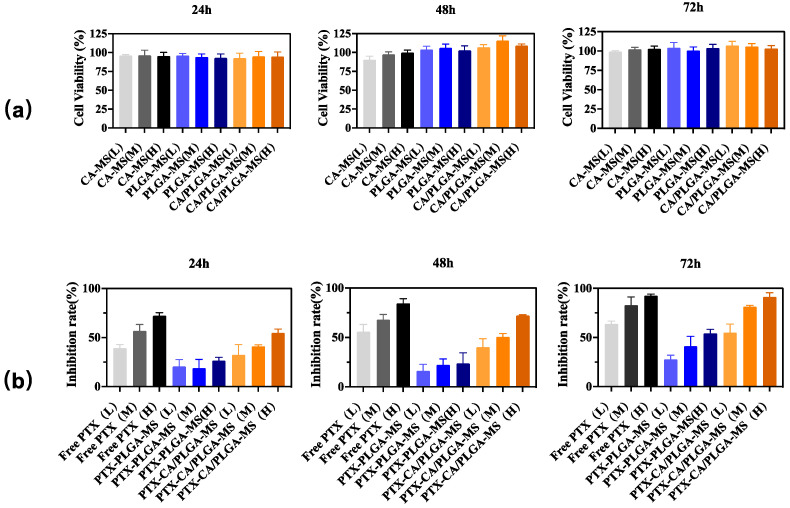
(**a**) HepG2 cells were used to perform in vitro cytotoxicity tests on blank microspheres CA-MS, PLGA-MS, and CA/PLGA-MS. (**b**) HepG2 cells were used to perform in vitro cytotoxicity tests on drug-loaded microspheres PTX-PLGA-MS and PTX-CA/PLGA-MS compared with Free PTX. L: low dosage; M: medium dosage; H: high dosage; CA-MS: single-layer alginate calcium MS; PLGA-MS: single-layer poly (lactic acid-co-glycolic acid) MS; CA/PLGA-MS: core-shell structured MS with an outer layer of alginate calcium and an inner layer of poly (lactic acid-co-glycolic acid) copolymer; PTX: paclitaxel; PTX-PLGA-MS: paclitaxel loaded into a single layer of poly (lactic acid-co-glycolic acid) MS; PTX-CA/PLGA-MS: paclitaxel into MS with a core-shell structure, with the outer layer being calcium alginate and the inner layer being poly (lactic acid-co-glycolic acid) copolymer. Results are presented as mean ± SD, n = 6. MS: Microspheres.

Figure 5 shows the CCK-8 assay results on the cytotoxic effects of CA-MS, PLGA-MS, CA/PLGA-MS, free PTX, PTX-PLGA-MS, and PTX-CA/PLGA-MS. Because both PLGA and alginate have been proven safe, no obvious cytotoxicity was observed in either group, indicating that the MS had good biocompatibility. Moreover, the low cytotoxicity indicates that the organic solvent in the MS was effectively removed by freeze-drying, resulting in very low solvent residue. Compared with PTX-PLGA-MS, because initial rapid release can provide high drug concentration, PTX-CA/PLGA-MS showed a high cell growth inhibition effect, which was similar to that of the free drug.

### 3.4. In Vivo Anti-Tumor Activity and Drug Release

**Figure 6:**
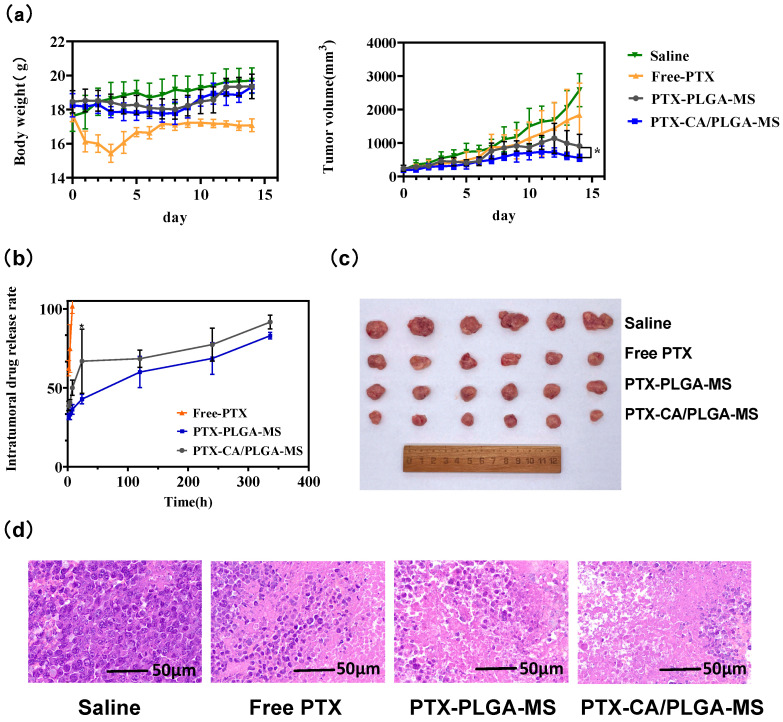
(**a**) Change in body weight and tumor volume from 0–15 d; data are presented as mean ± SD (n = 6); * *p* < 0.05. (**b**) Drug release profile for free PTX, PTX-PLGA-MS, and PTX-CA/PLGA-MS in tumors (n = 5). (**c**) Images of tumor masses excised from mice carrying HepG2 xenografts and treated for 14 d. (**d**) Histological features of liver tumor tissue after treatment. PTX-PLGA-MS: paclitaxel loaded into a single layer of poly (lactic acid-co-glycolic acid) MS; PTX-CA/PLGA-MS: paclitaxel into MS with a core-shell structure, with the outer layer being calcium alginate and the inner layer being poly (lactic acid-co-glycolic acid) copolymer; MS: Microspheres.

The changes in body weight and tumor volume in each group are shown in Figure 6a. The tumor volume in the saline group increased significantly, and the tumor volume on day 14 was approximately six times that of day 0, indicating that the tumor tissue activity was good. Although the free drug could inhibit the growth of tumor tissue to a certain extent, the data of 10–14 days show that the tumor volume markedly increased. This may be due to the fact that the free drug rapidly diffused from the injection site after injection, resulting in a short duration of drug action and therefore limited inhibition of tumor growth. When the tumor grows gradually, a low intratumoral drug concentration is unable to produce an effective anti-tumor effect. In contrast, PTX-PLGA-MS and PTX-CA/PLGA-MS exerted enhanced inhibitory effects on tumor proliferation. The difference in the tumor proliferation rates of the PTX-PLGA-MS and PTX-CA/PLGA-MS groups was not significant (*p* > 0.05) in the initial stage (within 5 d); however, in the later stage, the tumor inhibition effect of PTX-CA/PLGA-MS was better than that of PTX-PLGA-MS. This result may be due to the rapid release of PTX by PTX-CA/PLGA-MS in the early stage of injection, thus it could kill more tumor cells. PTX-PLGA-MS showed slow drug release after injection, thus having a delayed therapeutic effect. The body weight of the mice in the free PTX group decreased significantly, indicating that the drug had a somewhat strong toxic effect; therefore, intratumoral injection of the free drug could not significantly prevent drug-related adverse reactions. In contrast, mice in the PTX-PLGA-MS and PTX-CA/PLGA-MS groups did not lose significant body weight, indicating better safety. The in vivo drug release results are shown in Figure 6c. For the free-PTX group, free drug was not detected in the tumor 8 h after injection, indicating that the drug was rapidly distributed throughout the body, which also explains the strong toxic effects observed. As the drug release profile of PTX-CA/PLGA-MS had two stages, the drug release rate was higher within 24 h of dosage form injection, producing a stronger cytotoxic effect in a shorter time than simple PLGA MS. Moreover, the PLGA core continuously released the drug and maintained a certain drug concentration to achieve enhanced tumor inhibition. The results of H&E staining (Figure 6d) showed good tumor activity in the saline group. A small necrotic area was observed in the free PTX group, although the active tumor area was distributed substantially. Large necrotic areas were observed in both PTX-PLGA-MS and PTX-CA/PLGA-MS groups, indicating enhanced anti-tumor activity. 

## 4. Conclusions

In this study, PTX-loaded MS with a core-shell structure were prepared using a coaxial electrostatic spraying method, and their drug release profile and therapeutic potential was evaluated in vitro and in vivo. The results showed that a voltage of 8.0 kV yielded double-layered MS with good morphology, uniform particle size, and almost no solvent residue. The drug from PTX-CA/PLGA-MS was released in two stages. The first stage involved rapid release of the drug from the outer layer and rapid killing of tumor cells. In the second stage, the drug release from the core was relatively slow, thus showing sustained release characteristics to maintain a certain local drug concentration in the tumor. Compared with direct injection of the free drug and PTX-PLGA-MS, PTX-CA/PLGA-MS had a more significant tumor inhibitory effect. The findings of this study provide a new method and design for the preparation of multi-stage-controlled drug release microspheres for potential clinical application in therapeutics.

## Data Availability

The data are available from the corresponding author on reasonable request.
